# EasyDock: customizable and scalable docking tool

**DOI:** 10.1186/s13321-023-00772-2

**Published:** 2023-11-01

**Authors:** Guzel Minibaeva, Aleksandra Ivanova, Pavel Polishchuk

**Affiliations:** Institute of Molecular and Translational Medicine, Faculty of Medicine and Dentistry, Palacky University and University Hospital in Olomouc, Hnevotinska 5, 77900 Olomouc, Czech Republic

**Keywords:** High-throughput molecular docking, Distributed docking, Boron-containing compound docking, AutoDock Vina, Gnina

## Abstract

Docking of large compound collections becomes an important procedure to discover new chemical entities. Screening of large sets of compounds may also occur in de novo design projects guided by molecular docking. To facilitate these processes, there is a need for automated tools capable of efficiently docking a large number of molecules using multiple computational nodes within a reasonable timeframe. These tools should also allow for easy integration of new docking programs and provide a user-friendly program interface to support the development of further approaches utilizing docking as a foundation. Currently available tools have certain limitations, such as lacking a convenient program interface or lacking support for distributed computations. In response to these limitations, we have developed a module called EasyDock. It can be deployed over a network of computational nodes using the Dask library, without requiring a specific cluster scheduler. Furthermore, we have proposed and implemented a simple model that predicts the runtime of docking experiments and applied it to minimize overall docking time. The current version of EasyDock supports popular docking programs, namely Autodock Vina, gnina, and smina. Additionally, we implemented a supplementary feature to enable docking of boron-containing compounds, which are not inherently supported by Vina and smina, and demonstrated its applicability on a set of 55 PDB protein-ligand complexes.

**Supplementary Information:**

The online version contains supplementary material available at 10.1186/s13321-023-00772-2.

## Introduction

The primary objective during the early stages of drug discovery pipelines is the identification of promising hits. To accomplish this, high-throughput screening (HTS) is extensively applied to explore the chemical space and uncover initial hits. HTS enables the screening of libraries containing millions of compounds [1]. Although this may seem like a large number, it represents only a tiny fraction of the entire drug-like chemical space, which is estimated to contain approximately 10^36^ compounds [2]. The introduction of DNA-encoded combinatorial libraries has significantly expanded the coverage of chemical space and increased the number of compounds screened in a single campaign to the range of 10^9^–10^10^ [3–5]. However, DNA-encoded libraries are restricted by the types of chemical reactions suitable for coupling building blocks, and, thus, they cannot efficiently cover the entire chemical space.

Computational approaches may further extend the size of explored chemical space. They can be broadly categorized into two groups: virtual screening and de novo design. Recent studies have shown that virtual screening of ultra-large libraries is a promising approach for identifying highly active hits [6–8]. As a result, numerous academic and proprietary virtual libraries have been developed, containing up to 10^20^ compounds [9]. However, even these large libraries represent only a fraction of the entire chemical space, and their exhaustive screening is no longer feasible. De novo design approaches offer a solution to explore chemical space beyond the limitations of routine virtual screening. In de novo design, molecules are generated iteratively to satisfy specific criteria, in particular docking score. This allows for adaptive exploration of regions in chemical space likely to contain promising hits, without exhaustively enumerating the entire accessible chemical space. These approaches have demonstrated high efficacy in hit discovery, particularly in libraries containing over 10^10^ compounds [10]. In such campaigns, the number of docked molecules can reach millions. In both virtual screening and de novo design scenarios, there is a need to efficiently dock a large number of molecules, ranging from millions to billions, within a single campaign.

In order to advance the field of structure-based drug design, the development of fast, convenient, and reliable computational tools capable of efficiently docking millions of molecules within a reasonable timeframe is required. Several tools have been created to address this need, including Vina MPI [11], VirtualFlow [6], DockStream [12], DOCK3.7 [13], ChemFlow [14]. These tools enable distributed docking on clusters; however, they typically offer only a high-level interface and lack easy integration into other programs. This complicates their incorporation into developing approaches and software based on high-throughput docking. To address these limitations, DockString was developed as a Python module, providing a convenient interface for docking of individual ligands [15]. This offers greater flexibility for the development of customized applications. However, DockString does not support distributed computations, requiring users to create their own distributed workflow based on this module.

To overcome these challenges, we have developed a novel docking tool capable of performing calculations using either a single server or multiple servers within a network. This tool can be invoked from the command line or imported as a Python module, making it suitable for the development of further applications based on large-scale molecular docking. The current implementation of the tool supports AutoDock Vina [16], gnina [17] and smina [18] (as a component of gnina), and we suppose it can easily accommodate the integration of additional custom docking programs. As an additional feature we implemented the special protocol of docking of boron-containing compounds which cannot be processed natively by Vina and smina.

## Implementation

**Fig. 1:**
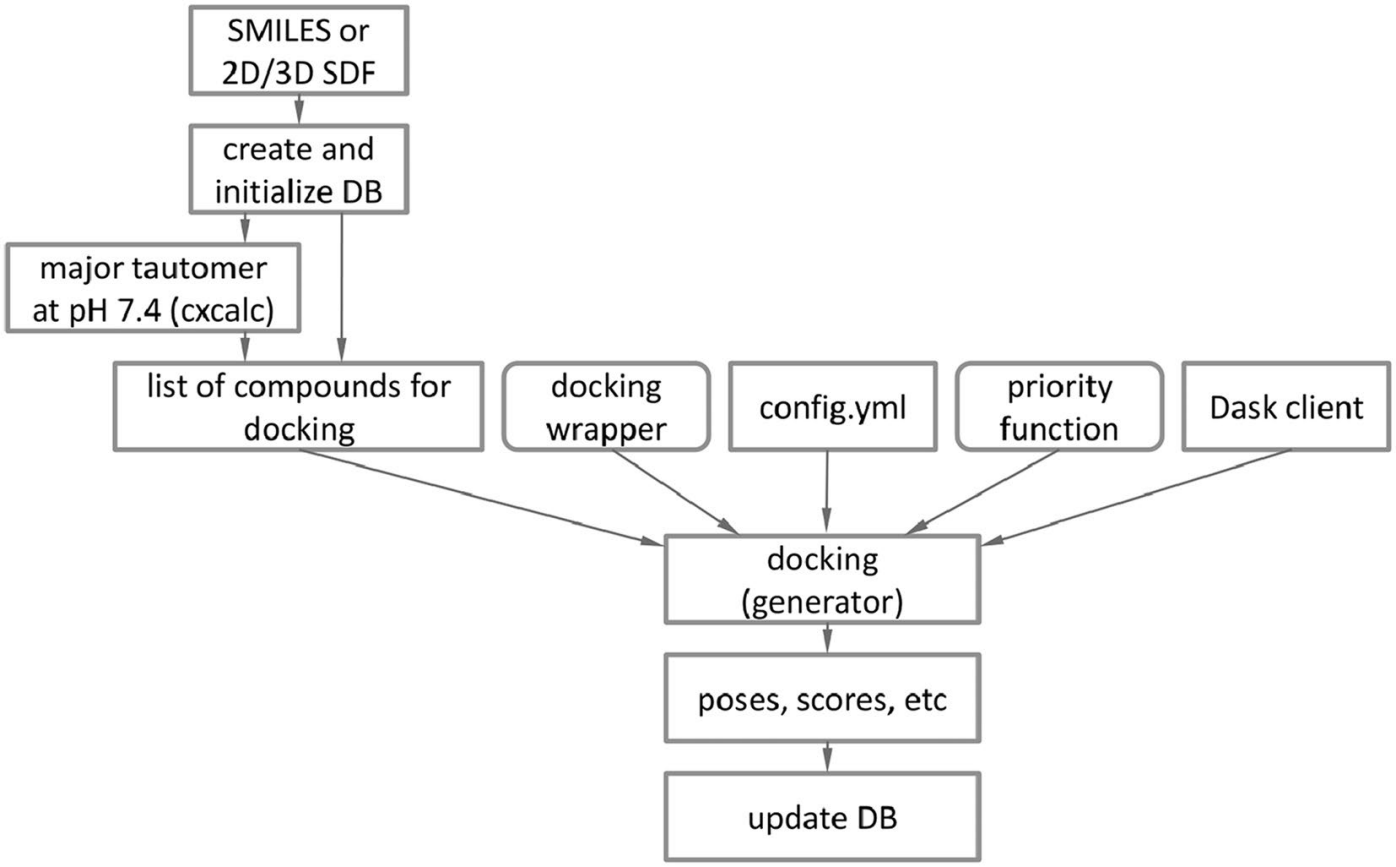
A high-level representation of the EasyDock workflow. Input molecules are stored in a database, optionally protonated and submitted to the main docking function which takes other docking settings as additional input parameters. Rounded rectangles designate customizable functions to introduce a custom docking program

The EasyDock module, implemented in Python 3, follows the workflow depicted in Fig. 1. Input to the module can be provided as SMILES or 2D/3D molecules in SDF format. If 3D structures are provided, they will be used as initial conformations for the docking process, otherwise, 3D embedding will be performed by RDKit. This enables the use of alternative conformer generators without explicitly integrating them into EasyDock. An SQLite database is created and populated with input molecules. All input arguments are also stored in the database that enables simple continuation of interrupted calculations. Optionally, the module can employ Chemaxon cxcalc utility to obtain major tautomers at pH 7.4. If molecules have been previously protonated, this step can be disabled, and the input molecules will be used as-is for docking.

The docking process is carried out by a generator function (Fig. 1), which takes the following inputs: (i) a list of molecules, (ii) a docking function that wraps a specific docking program and implements all logic including ligand preparation, docking itself and post-processing of docking output, (iii) a YAML configuration file (config.yml) containing values for all other arguments of the docking wrapper function, and (iv) an optional function that estimates the docking priority of individual molecules to optimize the overall running time. For each molecule, the generator yields a molecule name and a dictionary of output values (poses, scores, etc.), which are subsequently stored in the database. The docking generator can be imported and used in a third-party Python software development.

For single-machine calculations, the docking generator only requires the specification of the number of CPU cores. By default, it utilizes the multiprocessing module in Python to execute docking on multiple CPU cores. To perform distributed docking across multiple machines, the Dask Python library is employed. Dask creates a virtual cluster comprising individual workers distributed over a physical cluster or network of computational nodes. Importantly, Dask does not depend on any specific cluster scheduler, such as PBS or SLURM. To enable distributed computations, the user must first set up and initiate a Dask cluster before running the docking process. This can be accomplished with a single command executed from the command line. Subsequently, the docking program can be invoked by supplying the IP address of a parent node within the Dask cluster. The molecules are then sequentially submitted to the individual workers, and the results are gathered and stored in the database as they become available.

To customize EasyDock and enable support for other docking programs, one has to implement a docking wrapper function that takes a molecule and a configuration file as inputs. This wrapper function should return the molecule name and a dictionary of output values, which will be used to update the database. If the intention is to employ multiple cores for docking a single molecule, the wrapper function should internally launch a console script; otherwise an error will occur due to the Python Global Interpreter Lock.

Optionally, users have the opportunity to implement and supply a custom function responsible for estimating the docking priority of individual molecules. Higher priority should be assigned to molecules with longer docking runtimes. This approach reduces time wasted on completing computations because if one molecule is docked for a long time and it is the last one in the list, all other computational nodes will stand idle until docking of this molecule will be finished. By default, the number of rotatable bonds serves as a proxy for the priority function. The greater the number of rotatable bonds, the higher the docking priority. However, we recommend utilizing our custom priority function, which offers a more accurate estimation of docking runtime and is better suited for prioritizing the docking of molecules. Further details can be found in the Results and discussion section.

The current implementation of EasyDock supports two docking programs, namely AutoDock Vina and gnina. The scoring functions of smina are accessible through gnina interface. Each program has its own wrapper function, which performs the docking for individual molecules. The function consists of several steps:

1. Ligand preparation:
2. If a non-3D structure is provided, the input molecule undergoes 3D embedding using RDKit.
3. to address the docking of boron-containing compounds, boron atoms are substituted with carbon atoms. This workaround is necessary because boron atoms are not parameterized in Vina and smina, making it impossible to dock such compounds. Although a simplification, this replacement is reasonable due to the similar atomic properties of boron and carbon. We investigated this approach and confirmed its validity. Further details are provided in the Results and discussion section.
4. the molecule is converted to the PDBQT format using the Meeko module (https://github.com/forlilab/Meeko).
5. Docking process. For Vina docking, we utilize version 1.2.3, which includes Python bindings. Both Vina and gnina are invoked from the shell, providing input files and parameters. The docking of each molecule can be executed on a single core or multiple cores, depending on the chosen configuration.
6. Output parsing. The output PDBQT file is parsed, and the top-scored pose is converted to the MDL Mol format. If necessary, corresponding carbon atoms are replaced back with boron atoms during this conversion procedure.

### Dask library

Since Dask is not widely adopted by the chemoinformatic community for parallelization of tasks we briefly summarized its features and compared Dask with other tools.

Dask [19] is a Python library composed of two major parts: dynamic task scheduling through creation of a dynamic computational graph and “big data” collections supporting parallel processing of arrays and dataframes. The latter is more relevant for data analysis tasks using numpy and pandas. We chose Dask for implementation of docking parallelization because it suggests an easy programming interface and manages the distribution and scheduling of tasks onto computational nodes on its own. Its interface is very similar to the standard multiprocessing Python module if one needs to run in parallel multiple independent calculations, like docking of many compounds (an embarrassingly parallel task). It takes few lines of code to add support of parallel execution using Dask. Dask can be run over different schedulers (SLURM, PBS, etc.) and it can be also run over an arbitrary network of servers trough SSH connections. Dask also supports a dashboard to track the progress of calculations and node loading. We encountered only one issue related not to programming with Dask but to setup of the in-house cluster to effectively use Dask on multiple nodes. Dask uses file descriptors for intercommunication between nodes and therefore the allowed number of simultaneously opened file descriptors should be set accordingly. Overall, Dask is a mature project with good documentation and a relatively large community.

Message-Passing Interface (MPI) is a popular technology to run parallel tasks. In comparison to Dask it requires more low-level programming and is harder to learn. However, it offers better optimization of running tasks over multiple computational nodes with greater programming efforts.

Spark is similar to Dask. It provides a high-level programming interface and can be scaled to thousands of nodes. Spark is fundamentally an extension of the Map-Shuffle-Reduce paradigm while Dask supports arbitrary computational graphs by design. Spark is written in Scala and while there are ways of using Spark with Python, it is much more straightforward to use Dask, which is Python-native.

HyperQueue (https://github.com/It4innovations/hyperqueue) is a promising alternative of Dask which is worth to mention. It is a result of efforts on investigation and optimization of Dask scheduling model and overheads [20, 21]. HyperQueue has lower overheads than Dask. The development of HyperQueue was mostly focused on providing a command line interface to facilitate users to run parallel tasks using PBS/SLURM schedulers. However, it also offers Python interface similar to Dask but which is less mature. In future, if Python bindings of HyperQueue will be developed more extensively it may replace Dask, in particular in cases where one needs to run multiple calls of a function over a large number of instances.

### Protein preparation

To perform docking studies a user should submit a prepared protein structure. In this study we prepared receptors by the Dock Prep protocol implemented in Chimera [22]: missed side chains and sequences were remodeled using a Dunbrack rotomer library [23] and MODELLER [24], respectively, hydrogens were added considering pH 7.4 and solvent molecules were removed. Molecules were converted to PDBQT format using the prepare_receptor4.py utility from Autodock Tools. The grid boxes were determined from ligand coordinates. The center of a grid box is calculated as a geometric center of a ligand and the size of a box is calculated by adding of 7Å to minimum and maximum coordinates of ligand heavy atoms. The prepared protein structures and grid boxes are available in the repository https://github.com/ci-lab-cz/docking-files.

## Results and discussion

In this section we will describe two major features of the tool. The first one is the ability to dock boron-containing compounds, which is not possible with some of the integrated programs (Vina and smina). The second feature is the ability to run docking on a distributed infrastructure and we will describe its optimization and efficiency.

### Docking of boron-containing compounds

Application of boron-containing compounds in drug discovery projects is gradually increasing due to the ability of boron functional groups to form covalent and strong hydrogen bonds, modulate pharmacokinetics, drug resistance, etc. [25, 26]. Many popular docking programs do not support docking of boron-containing compounds by default. To overcome this limitation, we implemented a previously suggested protocol involving the substitution of boron atoms with carbon atoms prior to docking [27–30] followed by the revert replacement afterward. While this approach may appear artificial, it holds promise due to the similar atomic properties exhibited by boron and carbon.

To validate the hypothesis, we conducted a redocking study involving 55 non-covalent protein-ligand complexes that incorporated boron-containing compounds. These complexes were selected from the Protein Data Bank. Complexes had to have X-ray resolution of less than 2.5 Å and a ligand molecular weight of less than 500. Non-covalent binding was checked by visual inspection of complexes. We also omitted complexes with carborane-containing ligands because they have non-standard valence of atoms that cause errors in RDKit which is used for manipulation with molecular structures. For the sake of reference, all corresponding PDB codes and the associated redocking statistics are provided in Additional file 1: Table S1.

In the initial phase, we conducted docking experiments on the selected compounds using gnina. This docking program was chosen due to its ability to natively handle boron-containing compounds. We compared the results obtained from gnina docking of the original compounds to those obtained when boron atoms were replaced with carbon atoms. For the docking process, we set exhaustiveness to 32 and employed rescoring with default_ensemble or dense_ensemble. The protonation of compounds was performed using Chemaxon, as previously described.

Our analysis revealed that default_ensemble was not able to reproduce docking poses of boron-containing compounds with a reasonable accuracy (RMSD ≤ 2Å), only poses for 10 complexes were reproduced (18%), whereas dense_ensemble reproduced poses for 36 complexes (65%). Replacement of boron atoms with carbons only slightly affected the accuracy, which was increased, 12 poses for default_ensemble (22%) and 37 poses (67%) for dense_ensemble. We do not have explanation why default_ensemble performed poor. However, performance of dense_ensemble without and with boron replacement closely align with the general performance of gnina, which has been reported to range from 69 to 72% accuracy [17]. These results demonstrate that gnina treats boron and carbon atoms similarly and indirectly supports the hypothesis that boron and carbon atoms exhibit similar properties and can be interchangeable in docking simulations to some extent.

**Fig. 2:**
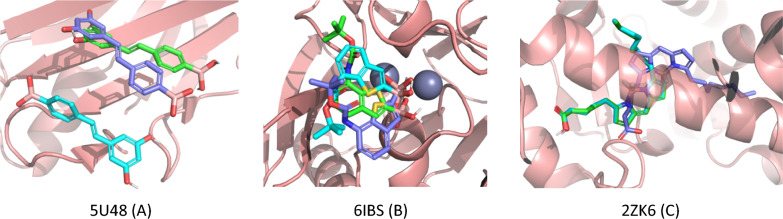
Native (blue) and unsuccessful (RMSD > 2Å) redocking poses obtained by Vina (green) and gnina dense_ensemble (cyan)

Next, we implemented the suggested protocol for automatic usage with Vina and smina. In both cases, we set an exhaustiveness value to 32, and for smina, we employed the Vinardo scoring function [31]. Out of the 55 ligands, Vina successfully reproduced the poses of 31 ligands (56%), while smina reproduced 30 ligands (54%). The result obtained with Vina corresponds well to the general accuracy of 58% reported previously [17]. Vina, smina and gnina mainly agree and disagree on the same ligands and complexes. Several failed complexes were associated with shallow binding sites. For instance, ligands that were co-crystallized on the surface of beta-sheets of Transthyretin are widely exposed to water and featured a limited number of specific interactions (e.g., 5U48, 5U4C, 5U4E) (Fig. 2A). Similarly, there were cases where the binding site was widely open, and the ligand tail was significantly exposed to water (e.g., 5LMD, 6IBS, 6JN6, 6L40, 6Q2Y, 6Q30) (Fig. 2B). In some cases, the ligand itself was large and highly flexible (e.g., 2ZK6) (Fig. 2C). Therefore, many of the poorly docked poses can be attributed to inherent issues in docking approaches and are not specific to boron-containing compounds or the suggested protocol. Based on these observations, we conclude that the proposed protocol for docking boron-containing compounds, which involves the replacement of boron atoms with carbon atoms, is applicable. However, researchers should exercise additional caution when working with such compounds.

### Customizing a function setting a priority of docking of individual molecules

To address the issue of potential wasting of computational resources, where some molecules may take excessively long time to dock while other computational nodes remain idle, we suggested to prioritize docking of molecules based on their estimated docking time. As an obvious estimator, the number of rotatable bonds can be used. In order to evaluate different estimators and estimate docking run times, we conducted a study.

We collected a set of 2.13 million compounds from ChEMBL (version 30) and calculated their molecular weights, finding that the 95th percentile was at 700 Da. We chose this threshold as a maximum value for typical bioactive molecules and selected a random subset of 10,000 molecules with molecular weights below 700 Da for further analysis. These molecules were docked into the CDK2 receptor (PDB: 2BTR) using AutoDock Vina with an exhaustiveness value of 8. CDK2 protein was chosen as one of the frequently used targets in benchmarking of docking programs [15]. 2BTR structure has a resolution 1.85Å and has no missing residues or other issues within the binding site, thus it is preparation was easy. On a single core, the median run time for the docking process was 190 s, with an average of 305 s. Among the 10,000 molecules, there were 1,217 instances which docking time exceeded 10 min.

For this set of 10,000 molecules, we calculated various physicochemical parameters including the number of H-bond donors (HBD) and acceptors (HBA), the number of rotatable bonds (RTB), molecular weight (MW), the number of rings (num rings), the number of heavy atoms (HAC), topological polar surface area (TPSA), and lipophilicity (logP). We then analyzed the correlation between these parameters and the docking run time. The highest correlating parameters with docking run time were molecular weight (R = 0.777), the number of heavy atoms (R = 0.784), and the number of rotatable bonds (R = 0.764). Other parameters exhibited correlation coefficients below 0.5 (Table 1). Considering the high correlation between molecular weight and the number of heavy atoms (R = 0.972), we selected the number of rotatable bonds and the number of heavy atoms as the two parameters for further studies.

**Table 1:** Pearson correlation (R) between physicochemical parameters and docking time (Autodock Vina) estimated by 10,000 molecules randomly chosen from ChEMBL30

	HBA	HBD	MW	HAC	RTB	logP	TPSA	Number of rings	Docking time
HBA	1	0.298	0.544	0.539	0.341	− 0.205	0.759	0.327	0.433
HBD	0.298	1	0.258	0.251	0.304	− 0.282	0.704	− 0.072	0.355
MW	0.544	0.258	1	0.972	0.596	0.467	0.51	0.587	0.777
HAC	0.539	0.251	0.972	1	0.595	0.476	0.501	0.649	0.784
RTB	0.341	0.304	0.596	0.595	1	0.258	0.386	− 0.036	0.764
logP	− 0.205	− 0.282	0.467	0.476	0.258	1	− 0.33	0.423	0.298
TPSA	0.759	0.704	0.51	0.501	0.386	− 0.33	1	0.113	0.494
Number of rings	0.327	− 0.072	0.587	0.649	− 0.036	0.423	0.113	1	0.279
Docking time	0.433	0.355	0.777	0.784	0.764	0.298	0.494	0.279	1

HBA: the number of H-bond acceptors; HBD: the number of H-bond donors; MW: molecular mass; HAC: the number of heavy atoms; RTB: the number of rotatable bonds; logP: lipophilicity; TPSA : topological polar surface area

To develop a simple and efficient estimator, we trained a linear model using the two selected parameters, namely the number of rotatable bonds and the number of heavy atoms. The dataset of 10,000 molecules was randomly split into a training set (90%) and a test set (10%). For the training set, we constructed models using 5-fold cross-validation, which was repeated five times to obtain an average estimate. Here, we present polynomial models with a degree of 2 (Table 2), as further increasing the degree did not significantly enhance the model performance. The results indicated that individual models based on the number of rotatable bonds or the number of heavy atoms exhibited moderate predictability (R^2^_test_ = 0.61–0.79). However, when these parameters were combined, the resulting model demonstrated high predictive ability (R^2^_test_ = 0.93) (Table 2). Equation 3, derived from this model, was implemented in EasyDock as an estimator of docking priority for individual molecules when Vina is selected as the docking program.

**Table 2:** Statistical parameters of linear models predicting docking time (Autodock Vina)

Equation number	Parameters	Equation	R^2^_CV_	R^2^_test_
1	HAC	time (s) = 624.144–63.215 × HAC + 1.735 × HAC^2^	0.765	0.789
2	RTB	time (s) = − 68.856 + 58.457 × RTB + 1.321 × RTB^2^	0.594	0.606
3	HAC, RTB	time (s) = 465.979–59.714 × RTB − 0.375 × RTB^2^ − 36.723 × HAC + 0.745 × HAC^2^ + 3.48 × RTB × HAC	0.925	0.926

Since the relationship between docking run time and molecular properties may vary for different docking programs and scoring functions there may be a need to develop a specific priority function. However, the developed priority function may be applicable to other programs to some extent. To assess the applicability of the suggested priority function, we conducted an investigation using gnina docking. We docked the test set of 1000 molecules with gnina, employing the following settings: scoring–default, cnn_scoring–rescore, cnn-default_ensemble or dense_ensemble. The docking calculations were performed exclusively on CPUs, with each molecule docked on a single core.

Surprisingly, we observed a high correlation between the predicted and observed run times: for both default_ensemble (R^2^(Pearson) = 0.882 and R^2^(Spearman) = 0.763) and dense_ensemble, (R^2^(Pearson) = 0.858 and R^2^(Spearman) = 0.652). Although the performance varied, the correlation was sufficiently strong to support the hypothesis that Eq. 3 may have broader applicability in ranking molecules based on their docking run times. Consequently, we implemented the same priority function (Eq. 3) for docking with gnina and smina. However, it should be emphasized that the implemented model is not universally applicable. For other docking programs, scoring functions, or different setups (such as utilizing GPUs), it may be necessary to develop a custom priority function to achieve optimal results.

### Scalability and general performance

To assess the computational efficiency and scalability of EasyDock, we randomly selected 5000 molecules from ChEMBL (version 30) with a molecular weight below 700. These molecules were distinct from those used to develop the priority function described earlier. Prior to docking, the molecules were converted to their major tautomers at pH 7.4 using the cxcalc Chemaxon utility [32]. Although this step is performed by EasyDock by default, we disabled it in order to avoid overhead and obtain more precise measurements of docking performance. The docking process was conducted using Autodock Vina with an exhaustiveness value of 8, and the target receptor was the CDK2 protein obtained from the 2BTR PDB complex. The experiments were performed on an in-house computational cluster comprising nodes equipped with Xeon CPU E5-2650@2.00 GHz processors, featuring 32 cores and 48 GB of memory. Nodes were interconnected by a 10GB network that may also affect overall performance.

Initially, we examined whether the application of the suggested priority function would lead to a reduction in docking time. Tests were conducted using 20 nodes, with each molecule docked on a single core, and a speedup of approximately 10% was observed (Table 3). Subsequently, we optimized the number of molecules processed on a single server and the number of cores allocated for docking of each molecule. When docking was performed on 20 nodes, shorter run times were observed with a greater number of cores per molecule. While slight overbooking did not significantly impact run times, we selected the following setup for subsequent runs as it resulted in the shortest run time: 8 molecules per server and 5 cores per molecule (Table 3). A control experiment using this setup, with molecules selected for docking in a random order, demonstrated a 1.55% slower run time. Although this difference is not substantial, it remained consistent across repeated runs. Thus, the application of the priority function had a noticeable and measurable effect, even with an optimal setup regarding the number of molecules and cores per server. This confirms the applicability of the suggested priority function in reducing docking run times.

We evaluated scalability of docking using the Dask library and compared the results with the native Python multiprocessing module. The native module exhibited smaller overheads for dispatching molecules for docking and outperformed the Dask-based implementation by 3.4% on a single server. However, the scalability of the Dask library was commendable, as increasing the number of nodes only slightly increased the overhead. For docking using 20 nodes, the overhead was 4.6%.

**Table 3:** Performance of docking of 5000 ligands to CDK2 (2BTR) with Autodock Vina using different number of computational nodes

Number of computational nodes (parallelization)	Total number of cores	N workers per node	N cpu per molecule	Wall time	Speed up
1 (multiprocessing, random priority)	32	8	5	7 h 4 m	1
1 (dask)	32	8	5	7 h 19 m	0.966
2 (dask)	64	8	5	3 h 39 m	1.936
5 (dask)	160	8	5	87 m 43 s	4.833
10 (dask)	320	8	5	44 m 8 s	9.607
20 (dask, random priority)	640	32	1	29 m 37 s	14.32
20 (dask)	640	32	1	26 m 45 s	15.85
20 (dask)	640	16	2	23 m 43 s	17.88
20 (dask)	640	16	3	23 m 21 s	18.16
20 (dask)	640	8	4	22 m 19 s	19.00
20 (dask)	640	8	5	22 m 14 s	19.07
20 (dask, random priority)	640	8	5	22 m 35 s	18.77

### Comparison with other programs

An overview of available automated docking tools is presented in Table 4. While some tools are designed to work with specific docking programs, many of them claim to offer extensibility with other programs, similar to EasyDock. Autodock Vina and its derivatives emerge as the most commonly integrated docking programs. EasyDock, in addition to Autodock Vina, also integrates gnina, which utilizes modern 3D convolutional neural networks and exhibits improved performance compared to Vina, albeit with higher computational requirements.

Most automated docking protocols offer 3D embedding of initial structures. Protonation of ligands is typically performed using OpenBabel, Epik, or Chemaxon cxcalc utility. DockStream and ChemFlow provide tautomer and stereoisomer enumeration capabilities. DockString generates consistent random stereoisomers but does not handle tautomers, whereas VirtualFlow generates major tautomers but lacks stereoisomer generation. EasyDock automatically generates major tautomers at pH 7.4 and consistent random stereoisomers.

Many of these tools support distributed computing using common schedulers such as PBS, SLURM, and others. However, they do not necessarily provide an API for seamless integration into further developing software. While DockString offers a Python API, it does not support distributed computing. EasyDock distinguishes itself by supporting distributed computing across various network devices without reliance on a specific scheduler. Furthermore, EasyDock provides a Python API, facilitating straightforward integration into third-party software.

**Table 4:** The list of freely available automated docking protocols

Program name	Year	Supported docking programs	Input	Protonation	3D embedding	Stereoisomers/ tautomers	Parallel computing	Ref	Repository link
Vina MPI	2013	vina	pdbqt				MPI	[11]	
VirtualFlow	2020	vina qvina qvina-w vina-carb smina autodockFR		cxcalc	chemaxon openbabel	A major tautomer (cxcalc)	SLURM Moab TORQUE PBS	[6]	https://github.com/VirtualFlow/VFVS
DockStream	2021	vina glide gold hybrid rDock		epik	corina ligprep omega rdkit	Enumeration of tautomers and stereoisomers	Across cores	[12]	https://github.com/MolecularAI/DockStream
DOCK	2021	DOCK	3D molecules				SGE PBS SLURM	[13]	
DockString	2022	vina	SMILES	openbabel, pH = 7.4	rdkit	Consistent random stereoisomer (rdkit)	A single mol dock can use multiple cores	[15]	https://github.com/dockstring/dockstring
ChemFlow	2023	plants vina qvina smina	SMILES, 2D SDF	epic	cxcalc	Dominant tautomers (> 10% probability), enumerate stereoisomers (cxcalc)	PBS SLURM	[14]	https://github.com/IFMlab/ChemFlow
EasyDock	2023	vina gnina smina	SMILES, 2D/3D SDF	cxcalc, pH = 7.4 (optional)	rdkit if input is not 3D	A major tautomer (cxcalc, optional) consistent random stereoisomer (rdkit)	Across cores and network nodes (Dask)	This work	https://github.com/ci-lab-cz/easydock

EasyDock features:

- single server and distributed docking over nodes in a network, it does not depend on a particular scheduler (e.g. PBS, SLURM, etc.), Dask has its own scheduler to dispatch jobs.
- Python API to develop further applications based on docking.
- customizability of the module with other docking programs.
- automatic continuation of interrupted calculations.
- docking of boron-containing compounds using Vina and smina.
- automatic generation of a major tautomer and its protonation using Chemaxon cxcalc utility (optional).
- use of user-defined conformations as starting if 3D structures were supplied as input. This can be useful if one wants to sample ring conformation with programs other than RDKit.
- output is a single database. Data can be retrieved using ordinary SQL queries or a script provided within the module.
- the current implementation was tested using CPUs only, however if computational nodes are equipped with GPUs they may be used if this is supported by a docking program itself.

EasyDock limitations and remarks:

- stereoisomer enumeration is not implemented in EasyDock. In cases where the input molecule has undefined configurations of stereocenters or double bonds, a random but consistent stereoisomer will be generated. We recommend explicitly enumerating stereoisomers using built-in RDKit functions or the provided script gen_stereo_rdkit.py from the repository https://github.com/DrrDom/rdkit-scripts.
- the scalability of EasyDock to a larger number of nodes or workers has not been tested. It is possible that the overhead could increase, or writing to the database could become a rate-limiting step when scaling up.
- when performing docking of individual molecules, each molecule runs in a separate instance of the docking program. It is important to carefully estimate the memory required for docking a single molecule in order to choose an appropriate number of workers per computational node and avoid exceeding the total memory limit. For example, docking with gnina using dense_ensemble typically requires around 3 GB of memory per molecule. Therefore, on a node with 48 GB of memory, the maximum number of simultaneously processed molecules should be limited to 16 to ensure memory constraints are met.

## Conclusions

The EasyDock module incorporates an automated docking protocol that supports distributed computing and provides a Python interface. The protocol is designed to minimize user intervention. It takes input molecules and returns all outputs to a single database. Currently, EasyDock supports Autodock Vina, smina, and gnina. The specific protocol was implemented for the docking of boron-containing compounds in Vina and smina. This protocol demonstrated the ability to accurately reproduce poses of boron-containing ligands in redocking studies. The list of supported docking programs can be easily expanded to accommodate other programs. To optimize the docking process, we have developed a linear model that prioritizes the selection of compounds for docking. By employing this model, we could minimize the overall docking runtime in a distributed computing environment, outperforming random ordering approaches. The model was trained using Autodock Vina outputs, but as demonstrated with gnina, it can be applicable to other programs and scoring functions. Thus, it is recommended as a default choice for integrating other docking programs into EasyDock. The EasyDock tool is open-source and freely available to the scientific community. Its purpose is to facilitate virtual screening campaigns of large compound libraries and aid in the development of structure-based design tools using molecular docking techniques.

### Availability and requirements

Project name: EasyDock.

Project home page: e.g. https://github.com/ci-lab-cz/easydock.

Operating system(s): Platform independent.

Programming language: Python 3.

Other requirements: RDKit, vina, gnina, dask.

License: BSD 3-clause.

Any restrictions to use by non-academics: no limitation.

### Supplementary Information

**Additional file 1: Table S1. **Complexes of non-covalent boron-containing ligands used for redocking with Vina, smina (Vinardo) and gnina.
