# Supplementary material for: EasyDock: customizable and scalable docking tool
Source: J Cheminform. 2023 Nov 1;15:102. doi: 10.1186/s13321-023-00772-2 (PMC10619229; doi:10.1186/s13321-023-00772-2)
Supplement: Supplementary file 1 — Additional file 1: Table S1. Complexes of non-covalent boron-containing ligands used for redocking with Vina, smina (Vinardo) and gnina. [file 13321_2023_772_MOESM1_ESM.docx]

**EasyDock: customizable and scalable docking tool**

Guzel Minibaeva, Aleksandra Ivanova, Pavel Polishchuk*

Institute of Molecular and Translational Medicine, Faculty of Medicine and Dentistry, Palacky University and University Hospital in Olomouc, Hnevotinska 5, 77900 Olomouc, Czech Republic

pavlo.polishchuk@upol.cz

**Additional file**

Table S1. Complexes of non-covalent boron-containing ligands used for redocking with Vina, smina (Vinardo) and gnina.

|  | RMSD | | | | | | uniprot | Protein name | Organism |
| --- | --- | --- | --- | --- | --- | --- | --- | --- | --- |
| PDB iD | vina | smina (vinardo) | gnina (dense ensemble) | gnina (dense ensemble, boron replacement) | gnina (default ensemble) | gnina (default ensemble, boron replacement) |  |  |  |
| 6PHT | 1.502 | 1.814 | 1.669 | 2.181 | 1.669 | 1.661 | A0A0J7JFD7 | Acetoin utilization deacetylase AcuC | Marinobacter subterrani |
| 6Q55 | 1.855 | 1.615 | 1.76 | 1.784 | 1.76 | 1.784 | A0A0S4TJL4 | Beta-Casp domain-containing protein | Cryptosporidium hominis |
| 6QOU | 3.006 | 3.137 | 1.95 | 1.942 | 0.589 | 4.179 | B1MDI3 | tRNA (guanine-N(1)-)-methyltransferase (EC 2.1.1.228) | Mycobacteroides abscessus |
| 6QQR | 2.995 | 3.041 | 0.268 | 4.584 | 0.268 | 2.998 | B1MDI3 |  |  |
| 6IBS | 4.061 | 6.28 | 3.652 | 5.216 | 4.837 | 4.212 | C7C422 | Metallo-beta-lactamase type 2 (EC 3.5.2.6) | Klebsiella pneumoniae |
| 6IBV | 0.459 | 4.981 | 0.451 | 0.448 | 4.884 | 4.888 | C7C422 |  |  |
| 6Q2Y | 5.414 | 5.232 | 5.368 | 5.346 | 5.368 | 1.598 | C7C422 |  |  |
| 6Q30 | 3.887 | 4.755 | 4.749 | 1.875 | 4.749 | 1.438 | C7C422 |  |  |
| 6V1M | 0.753 | 0.781 | 0.775 | 0.777 | 0.775 | 0.777 | C7C422 |  |  |
| 7EUN | 1.023 | 1.389 | 0.849 | 0.665 | 0.849 | 0.665 | D2Z025 | N(omega)-hydroxy-L-arginine amidinohydrolase (EC 3.5.3.25) | Streptomyces lavendulae |
| 3MNU | 1.306 | 1.457 | 1.295 | 1.3 | 1.295 | 1.42 | P00918 | Carbonic anhydrase 2 (EC 4.2.1.1) | Homo sapiens (Human) |
| 5LMD | 3.017 | 3.359 | 2.362 | 1.423 | 3.134 | 3.185 | P00918 |  |  |
| 6RVF | 1.199 | 1.305 | 1.492 | 1.18 | 3.106 | 3.145 | P00918 |  |  |
| 6RVK | 3.363 | 2.765 | 1.361 | 1.509 | 3.34 | 0.841 | P00918 |  |  |
| 6RVL | 2.815 | 1.078 | 1.036 | 0.882 | 2.389 | 2.466 | P00918 |  |  |
| 6RW1 | 2.983 | 3.035 | 0.757 | 0.876 | 2.993 | 2.306 | P00918 |  |  |
| 6XVH | 1.065 | 4.033 | 0.743 | 0.758 | 0.743 | 0.758 | P00918 |  |  |
| 5U48 | 2.38 | 10.549 | 10.403 | 10.177 | 8.292 | 10.562 | P02766 | Transthyretin | Homo sapiens (Human) |
| 5U4C | 3.027 | 3 | 10.909 | 7.718 | 10.909 | 7.718 | P02766 |  |  |
| 5U4E | 3.986 | 3.066 | 7.897 | 7.935 | 3.395 | 7.871 | P02766 |  |  |
| 4HXQ | 1.964 | 1.261 | 1.435 | 1.811 | 1.595 | 1.516 | P05089 | Arginase-1 (EC 3.5.3.1) | Homo sapiens (Human) |
| 4IE1 | 1.385 | 2.047 | 1.657 | 1.553 | 2.204 | 2.26 | P05089 |  |  |
| 6QAF | 1.671 | 1.924 | 1.393 | 1.368 | 1.393 | 1.368 | P05089 |  |  |
| 6V7C | 1.014 | 1.129 | 0.795 | 0.876 | 1.79 | 1.766 | P05089 |  |  |
| 6V7D | 0.713 | 0.704 | 0.689 | 0.691 | 0.689 | 1.449 | P05089 |  |  |
| 6V7E | 0.589 | 0.572 | 0.598 | 0.601 | 0.598 | 0.601 | P05089 |  |  |
| 6V7F | 1.386 | 1.457 | 1.474 | 0.567 | 1.474 | 1.444 | P05089 |  |  |
| 7K4G | 1.078 | 0.598 | 0.638 | 0.51 | 1.96 | 1.907 | P05089 |  |  |
| 7K4H | 2.336 | 0.517 | 0.524 | 0.511 | 1.33 | 1.364 | P05089 |  |  |
| 7K4I | 0.827 | 1.911 | 0.826 | 0.822 | 1.643 | 1.637 | P05089 |  |  |
| 7K4J | 0.696 | 0.431 | 1.078 | 0.695 | 2.04 | 1.055 | P05089 |  |  |
| 7K4K | 0.993 | 1.328 | 2.05 | 2.092 | 2.05 | 2.092 | P05089 |  |  |
| 7KLK | 1.267 | 1.332 | 1.289 | 1.502 | 1.459 | 1.502 | P05089 |  |  |
| 7KLL | 2.094 | 1.038 | 1.306 | 1.372 | 1.794 | 1.372 | P05089 |  |  |
| 7KLM | 0.894 | 0.82 | 0.942 | 0.928 | 0.976 | 2.608 | P05089 |  |  |
| 8AUP | 0.559 | 1.374 | 1.356 | 0.514 | 0.587 | 0.514 | P05089 |  |  |
| 6UBO | 0.476 | 0.612 | 0.479 | 0.489 | 4.927 | 4.927 | P0A901 | Outer membrane lipoprotein Blc | Escherichia coli (strain K12) |
| 4XUD | 1.211 | 1.195 | 2.735 | 1.232 | 2.603 | 7.246 | P21964 | Catechol O-methyltransferase (EC 2.1.1.6) | Homo sapiens (Human) |
| 5OL3 | 7.641 | 8.182 | 2.154 | 1.259 | 2.154 | 1.987 | P25321 | cAMP-dependent protein kinase catalytic subunit alpha (PKA C-alpha) (EC 2.7.11.11) | Cricetulus griseus (Chinese hamster) |
| 7FLD | 7.776 | 7.775 | 1.285 | 1.238 | 1.285 | 1.238 | P33334 | Pre-mRNA-splicing factor 8 | Saccharomyces cerevisiae |
| 2ZK6 | 7.097 | 7.085 | 7.14 | 7.123 | 7.14 | 7.123 | P37231 | Peroxisome proliferator-activated receptor gamma (PPAR-gamma) (Nuclear receptor subfamily 1 group C member 3) | Homo sapiens (Human) |
| 1JX6 | 0.411 | 0.431 | 2.502 | 7.284 | 2.502 | 7.835 | P54300 | Autoinducer 2-binding periplasmic protein LuxP | Vibrio harveyi (Beneckea harveyi) |
| 4IE3 | 1.171 | 1.44 | 1.764 | 1.375 | 1.509 | 1.375 | P78540 | Arginase-2, mitochondrial (EC 3.5.3.1) | Homo sapiens (Human) |
| 4IXU | 1.588 | 3.147 | 2.414 | 2.632 | 3.705 | 4.172 | P78540 |  |  |
| 4IXV | 2.663 | 2.743 | 3.219 | 2.854 | 2.94 | 2.854 | P78540 |  |  |
| 6Q37 | 1.144 | 1.419 | 0.937 | 0.636 | 1.251 | 1.309 | P78540 |  |  |
| 6Q39 | 2.875 | 2.937 | 0.841 | 0.654 | 0.841 | 0.654 | P78540 |  |  |
| 7NOV | 0.751 | 0.441 | 3.982 | 5.567 | 6.202 | 6.23 | P9WIT9 | Ornithine carbamoyltransferase (OTCase) (EC 2.1.3.3) | Mycobacterium tuberculosis |
| 7NP0 | 4.128 | 0.575 | 4.336 | 3.473 | 4.135 | 0.543 | P9WIT9 |  |  |
| 3O0J | 3.188 | 2.998 | 3.216 | 5.621 | 5.673 | 5.701 | Q07343 | cAMP-specific 3',5'-cyclic phosphodiesterase 4B (EC 3.1.4.53) (DPDE4) (PDE32) | Homo sapiens (Human) |
| 5K6J | 1.031 | 0.738 | 1.403 | 4.335 | 1.403 | 1.381 | Q07343 |  |  |
| 6L40 | 5.797 | 5.718 | 6.024 | 6.014 | 5.444 | 6.512 | Q2YSF8 | ATP-dependent Clp protease proteolytic subunit (EC 3.4.21.92) (Endopeptidase Clp) | Staphylococcus aureus |
| 5HJA | 1.218 | 3.048 | 1.403 | 1.193 | 2.375 | 2.803 | Q6TUJ5 | Arginase (EC 3.5.3.1) | Leishmania mexicana |
| 7PUI | 8.842 | 9.179 | 1.743 | 2.289 | 8.829 | 8.834 | Q8IEE9 | choline-phosphate cytidylyltransferase (EC 2.7.7.15) | Plasmodium falciparum (isolate 3D7) |
| 6JN6 | 5.508 | 5.444 | 4.507 | 1.752 | 4.507 | 5.2 | Q9K2N0 | Beta-lactamase VIM-2 | Pseudomonas aeruginosa |
